# Serum levels of IL-6 and TNF-*α* correlate with clinicopathological features and patient survival in patients with prostate cancer

**DOI:** 10.1038/sj.bjc.6601814

**Published:** 2004-05-18

**Authors:** V Michalaki, K Syrigos, P Charles, J Waxman

**Affiliations:** 1 Department of Cancer Medicine, Faculty of Medicine, Imperial College of Science, Technology & Medicine, Hammersmith Campus Du Cane Road, London W12 ONN, UK; 2 Third Department of Medicine, University of Athens, 40 Kifisias and Arkadias, 115 25 Athens, Greece; 3 Kennedy Institute of Rheumatology, Faculty of Medicine, Imperial College of Science, Technology & Medicine, London, UK

**Keywords:** cytokines, IL-6, TNF-*α*, prostate cancer

## Abstract

Interleukin-6 (IL-6) and tumour necrosis factor-alpha (TNF-*α*) are important multifunctional cytokines involved in tumour growth and metastasis. In this study, we have measured serial levels of serum IL-6 and TNF-*α* in prostate cancer patients. A total of 80 patients with carcinoma of the prostate and 38 controls were studied. Three patient groups, with small bulk localised, large volume localised and metastatic prostate cancer, were assessed. Serum IL-6 and TNF-*α* levels were measured and correlated with clinicopathological variables and patient survival. Serial changes in these cytokines were also assessed and related to disease progression in 40 patients with recurrent prostate cancer. Serum IL-6 levels in patients with metastatic disease (9.3±7.8 pg ml^−1^) were higher than those in patients with localised disease (1.3±0.8 pg ml^−1^, *P*<0.001). Significantly elevated levels of TNF-*α* were found in metastatic disease (6.3±3.6 pg ml^−1^) compared with localised disease (1.1±0.5 pg ml^−1^, *P*<0.001). The levels of both cytokines were directly correlated with the extent of the disease. Serial analysis in 40 patients with recurrent tumours showed that both cytokines became elevated at the point of prostate-specific antigen progression. In conclusion, these results suggest that IL-6 and TNF-*α* correlate with the extent of disease in patients with prostate cancer and may be monitored in conjunction with other disease markers.

Prostate cancer is the most common cancer in men in the Western world and the second most common cause of male cancer deaths. The progression of prostate cancer to a metastatic state and to hormone independence is thought of as a multi-step process involving growth factors, hormones and cytokines (Bok and Small, 2002; Huss *et al*, 2003).

Although there are clear descriptions of the relationship of cytokines to the development and progression of many cancers, there are few published observations relevant to prostate cancer. Surprisingly perhaps for a tumour where there is such clear evidence of a primary hormonal basis to its development, there is emerging evidence of cytokine involvement. IL-6 and TNF-*α*, two cytokines with multiple and overlapping biological properties, are involved in prostate cancer development (Shariat *et al*, 2001; Smith *et al*, 2001).

This involvement of IL-6 at a cellular level with the processes of cancer control is reflected by the results of serum studies of cancer patients, where IL-6 may reflect prognosis and tumour load. Elevated IL-6 levels have been associated with advanced stage and metastasis-related morbidity (Adler *et al*, 1999; Nakashima *et al*, 2000; Wise *et al*, 2000). Thus, it has been recently reported that patients with metastatic ovarian cancer and patients with metastatic renal cell carcinoma have higher serum IL-6 levels than those without disseminated disease (Blay *et al*, 1992; Scambia *et al*, 1995). It has also been demonstrated that elevated IL-6 levels are associated with a poor prognosis in tumours such as non-small-cell lung cancer. The ontological role of IL-6 in this process is not known (De Vita *et al*, 1998).

Studies of IL-6 in prostate carcinoma provide support for the hypothesis that direct local production of IL-6 by malignant cells significantly contributes to elevated serum levels (Deeble *et al*, 2001).

IL-6 and TNF-*α* are considered the major mediators of a network of interactive signals. TNF-*α* is a pleiotropic cytokine, which like IL-6 has been shown to be associated with cancer progression. It is of interest that many androgen-insensitive prostate cancer cells are TNF-*α* insensitive. This may be because of the upregulation of a series of antiapoptotic genes involved in a network of paracrine and autocrine loops that modulate prostate carcinoma cell activity, and these include the nuclear factor-kappa*β* (NF-*κβ*) family of transcription factors (Muenchen *et al*, 2000).

In a series of 122 patients with renal cell carcinoma, Yoshida *et al* (2002) demonstrated that serum levels of TNF-*α* directly correlated with advanced stage grouping as compared with controls, and suggested that TNF-*α* could be useful in the early diagnosis of the disease. There has been a single study by Nakashima *et al* (1998), where TNF-*α* has been shown to be associated with prostate cancer progression. Serum TNF-*α* activity was positive in 76% of the patients with relapsed disease who had a significantly higher mortality rate than those with undetectable serum TNF-*α* levels.

In this study, we measured the serum levels of IL-6 and TNF-*α* in prostate cancer patients, using a highly sensitive ELISA kit, in an attempt to define their association with clinicopathological features and clinical outcome.

## MATERIALS AND METHODS

### Patients

A total of 80 men with prostate cancer (mean age 74.5±7.5 years) were included in this prospective study and compared with two normal control groups, consisting of 12 men presenting for prostate cancer screening who had no prior history of cancer or chronic disease, a normal digital rectal examination and a prostate-specific antigen (PSA) of less than 2.0 ng ml^−1^, which is in the PSA range with an estimated prostate cancer detection probability of less than 1% in the first 4 years after screening (Smith *et al*, 1996), and 26 patients with biopsy-proven benign prostatic hyperplasia (1).

The mean control age was 67.5±6.8 years. All patients had histological confirmation of the diagnosis of prostate cancer by needle biopsy or by transurethral resection of the prostate. Each patient was staged by clinical examination, bone scanning and computed tomography of the abdomen and pelvis. For the histologic grade the Gleason scoring was used. No patients had evidence of active infection or inflammatory disease.

To further evaluate the association between IL-6 and TNF-*α* and the progression of the disease, we assessed the serial changes of both cytokines in patients with recurrent disease. Therefore, at the time of the study, three samples from each patient with locally advanced or metastatic cancer were collected. The samples were chosen to represent the time points of presentation prior to treatment, the first evidence of PSA increase beyond the normal range and disease progression.

Out of 26 patients with localised disease, 12 were initially treated with radiation therapy or surgery, whereas all patients with locally advanced or metastatic disease were treated with endocrine therapy using a luteinising hormone-releasing analogue and flutamide. Neither patients with metastatic disease nor patients with localised disease were treated before serum collection.

### Sample collection and cytokine measurements

Blood samples were prospectively collected with the appropriate Ethical Committee permissions, from patients attending Oncology Outpatients Clinics at the Hammersmith Hospital. Each sample had been collected in a Vacutainer, (Becton Dickinson, Plymouth, UK) and serum was separated within 1 h of blood collection after spinning for 15 min at 1500 **g**. The serum was stored without preservative at −70°C and then thawed just prior to testing (2).

Serum IL-6 and TNF-*α* concentrations were determined using the commercially available enzyme-linked immuno-sorbent assay (ELISA), US (Ultra sensitive), kit supplied by Biosource International (CA, USA). The assays employ the quantitative sandwich enzyme immunoassay technique using recombinant human IL-6 and TNF-*α*, with antibodies raised against the recombinant proteins, respectively. The IL-6 ELISA assay has a performance sensitivity of less than 0.16 pg ml^−1^ with the minimum detectable level at 104 fg ml^−1^. The TNF-*α* assay has a performance sensitivity of less than 0.5 pg ml^−1^ and the minimum detectable level was 0.09 pg ml^−1^. Optical density was read with a microtiter plate reader by dual wavelength at 450 nm. All samples were assayed in duplicate.

Elevated serum levels of the studied cytokines were defined as being greater than the 95th percentile values in the healthy control group. This resulted in a cutoff value of 2.1 and 1.9 pg ml^−1^ for IL-6 and TNF-*α*, respectively.

### Follow-up

Follow-up data were obtained for all patients. Details of clinical progress and survival were obtained from Hospital and General Practice records and traced to Death Certificates where appropriate. Biochemical progression was described as that point when PSA levels exceeded 0.2 ng ml^−1^ on two or more occasions. The date of progression was assigned as the date of the first value greater than 0.2 ng ml^−1^.

The overall survival (OS) time was calculated for each patient to the nearest month, taken from the time of presentation to the time or death/last recorded follow-up. The median time of follow up was 48 months.

### Statistical analysis

The Kruskal–Wallis analysis of variance (ANOVA) was applied to evaluate differences in cytokine levels between unpaired and paired multiple group observations. Logistic regression analysis and the general linear model for repeated measurements were performed to assess the significance of changes in serial measurements of cytokines in individual patients at presentation, and relapse. Differences in the cytokines levels among clinical and pathologic features were tested by use of the Mann–Whitney test.

Spearman's rank correlation coefficient was used to compare the ordinal and continuous variables. The Kaplan–Meier method was used to calculate survival functions, and differences were assessed by log-rank analysis. Multivariate survival analysis was performed using the Cox proportional regression model. Significance was presumed at values of *P*<0.05.

Statistical analysis was performed with the Statistical Package for the Social Sciences (SPSS) statistical software package version 10.0 for Windows.

## RESULTS

### Clinical and pathologic characteristics

In all, 26 patients (33%) had localised small volume (T1, T2, N0, M0) cancers confined to the prostate, 14 patients (17%) had bulky (T3, T4, N0, M0) locally advanced disease and 40 patients (50%), had metastatic disease. The patients' characteristics are summarised in Table 1

**Table 1:** Patients' characteristics

	**Patients**	
	**No**	**%**
*Clinical stage*		
T1a	4	5
T1b+c	7	8.8
T2a	8	10
T2b	4	5
T2c	3	3.8
T3	9	11.2
T4	5	6.2
M1	40	50
		
*Gleason score*		
2–4	12	14
5–6	50	63
7–10	18	23

.

The Gleason score was 2–4 in 12 patients (14%), 5–6 in 50 patients (63%) and 7–10 in 18 patients (23%). The median PSA levels in patients with localised disease were 5.2 ng ml^−1^ (range 1.86–15.8), 19.2 ng ml^−1^ (range 8.6–96.7) and 113.5 ng ml^−1^ (range 23.7–450.2), in patients with locally advanced and metastatic disease, respectively (2).

### Baseline serum levels of cytokines in controls and prostate cancer patients

Both IL-6 and TNF-*α* were detectable in all control subjects and prostate cancer patients. In all of the prostate cancer patients, IL-6 and TNF-*α* levels were 5.6±6.7 and 4.3±3.6 pg ml^−1^, respectively, and significantly higher than the controls (1.1±0.6 and 1.2±0.4 pg ml^−1^, respectively, *P=*0.031 and 0.003) (Table 2

**Table 2:** Serum levels of IL-6 and TNF-*α* in all prostate cancer patients and controls according to stage

					**95% confidence intervals**	
		***n***	***P*-value^a^**	**Mean±s.d.**	**Lower bound**	**Upper bound**
IL-6 (pg ml^−1^)	P. cancer	80	0.031 (*vs* controls)	5.65±6.74	4.15	7.15
	(all)_					
	Localised	26	<0.001 (*vs* metastatic)	1.27±0.81	0.93	1.59
	Locally adv.	14	0.001 (*vs* metastatic)	3.50±2.88	1.82	5.16
	Metastatic	40	<0.001 (*vs* controls)	9.26±7.81	6.76	11.7
	Controls	12		1.13±0.63	0.73	1.53
	BPH	26		1.20±0.49	1.00	1.40
						
TNF-*α*(pg ml^−1^)	P. cancer	80	0.003 (*vs* controls)	4.30±3.69	3.47	5.12
	(all)_					
	Localised	26	0.03 (*vs* metastatic)	1.38±0.69	1.10	1.66
	Locally adv.	14	0.02 (*vs* metastatic)	3.90±3.43	1.90	5.87
	Metastatic	40	<0.001 (*vs* controls)	6.34±3.66	5.16	7.51
	Controls	12		1.08±0.55	0.73	1.43
	BPH	26		1.21±0.65	0.94	1.47

a Mann–Whitney *U* test.

).

When results from the three groups of patients with cancer were individually compared with the controls and patients with BPH, IL-6 levels were significantly higher in patients with metastatic disease (mean 9.3±7.8 pg ml^−1^, median 7.0 pg ml^−1^, *P*<0.001). Significantly elevated levels of TNF-*α* were found in patients with bulky locally advanced (mean 3.9±3.4 pg ml^−1^, median 2.2 pg ml^−1^) and metastatic disease (mean 6.3±3.6 pg ml^−1^, median 6.0 pg ml^−1^) as compared with controls (mean 1.1±0.5 pg ml^−1^, median 0.9 pg ml^−1^) (*P*=0.05 and *P*<0.001, respectively), but not in patients with localised small volume prostate cancer (Table 2).

When patients with metastatic prostate cancer were compared with both groups of patients with localised prostate cancer, there were significant differences in IL-6 and TNF-*α* levels among the groups. The group of patients with lymph node metastases or bone metastases had similar IL-6 and TNF-*α* levels (*P*=0.31 and *P*=0.28), which were higher than those in the group with localised disease (*P*<0.001).

Serum IL-6 and TNF-*α* levels were significantly elevated in patients with Gleason score >6 (mean 7.8±5.3 pg ml^−1^, median 4.0 pg ml^−1^, *P=*0.001 with respect to IL-6; mean 5.2±3.7 pg ml^−1^, median 5.1 pg ml^−1^, *P=*0.009 with respect to TNF-*α*).

Univariate analysis showed a positive correlation between disease stage and IL-6 and TNF-*α* levels (IL-6: correlation coefficient, 0.32; *P*<0.001; TNF-*α*: correlation coefficient 0.29; *P*=0.008). TNF-*α* levels were also correlated with PSA (correlation coefficient, 0.41; *P*<0.001).

To identify the variables of prognostic significance in all of the patients with prostate cancer, univariate analysis of each variable was performed in relation to the survival time. On univariate Cox proportional hazards regression analysis (Table 3

**Table 3:** Univariate and multivariate Cox regression analysis for predictors of survival in prostate cancer patients with localised disease

	**Univariate**		**Multivariate**		
	**Hazards ratio**	***P***	**Hazards ratio**	***P***	**95% CI**
Gleason score	10.53	<0.0001	8.98	<0.0001	2.92–28.75
IL-6^a^	3.6	0.031	2.1	0.053	097–5.45
TNF-*α*^a^	1.9	0.042			
PSA^b^	5.2	<0.01			

a TNF-*α* categorised as above or below the cutoff level of 1.9 pg ml^−1^.

**Figure 1:**
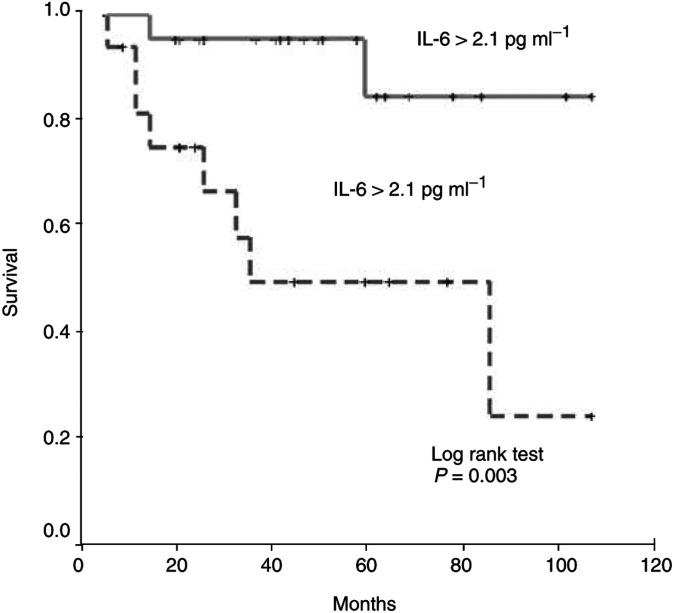
Disease-specific survival in patients with localised prostate cancer, stratified into groups above or below the cutoff IL-6 level of 2.1 pg ml^−1^.

IL-6 categorised as above or below the cutoff level of 2.1 pg ml^−1^.

b PSA was categorised as >or <100 ng ml^−1^.

), serum IL-6 (above the cutoff level of 2.1 pg ml^−1^) and serum TNF-*α* (above the cutoff level of 1.9 pg ml^−1^) were significant prognostic factors for survival, along with Gleason sum and serum PSA (categorised as >100 ng ml^−1^; Figure 1

).

**Figure 2:**
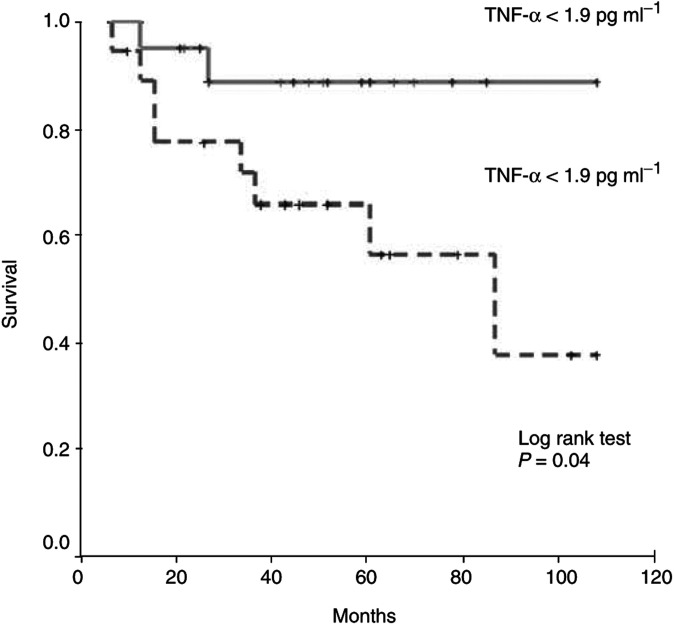
Disease-specific survival in patients with localised prostate cancer stratified into groups above or below the cutoff TNF-*α* level of 1.9 pg ml^−1^.

By the log-rank test, we found that patients with localised disease and serum IL-6 levels above the cutoff level (2.1 pg ml^−1^) had an increased probability of poor overall survival. Similarly, the same group of patients with serum TNF-*α* levels above the cutoff level (1.9 pg ml^−1^) had the worst outcome (*P*=0.04; Figure 2

).

However, the multivariate regression analysis failed to confirm these cytokines as significant independent prognostic variables for cancer-specific survival.

### Serial changes in levels of cytokines at presentation, PSA progression and symptom progression

**Figure 3:**
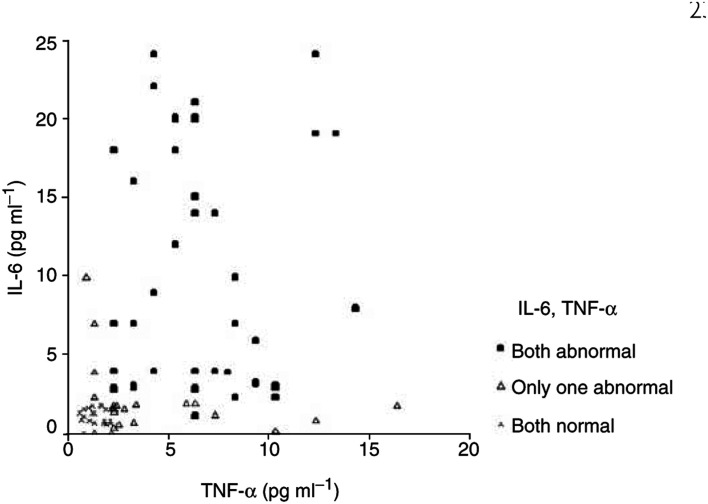
Graph of both IL-6 and TNF-*α* levels in 80 prostate cancer patients.

To further evaluate the association between IL-6 and TNF-*α* and the progression of the disease, we additionally assessed the serial changes of these cytokines in 40 patients with bulky locally advanced or metastatic cancer, at the time points of presentation prior to treatment, at the first time point when PSA started to rise and the point at which there was progression to symptomatic recurrent disease with bone pain or increased obstructive urinary symptoms (Figure 3

).

Analysis of the serial measurements revealed significant differences in both cytokines during the evolution of the disease. The levels of each cytokine were significantly higher at the time points of PSA progression compared with the baseline levels. All patients with symptomatic progressive disease had significantly higher levels of IL-6 (mean 9.3±7.0, *P*<0.01) and TNF-*α* (mean 6.2±3.6, *P*<0.01) than at presentation (mean 1.1±0.7, *P*<0.021 and 1.3±0.6, *P*<0.026 for IL-6 and TNF-*α*, respectively), or than at the point of biochemical relapse (mean 3.1±1.47, *P*<0.001 and 4.5±3.4, *P*<0.01 for IL-6 and TNF-*α*, respectively).

The univariate logistic regression analysis revealed that both cytokines were significant predictors for the evolution of the disease. Thus, considering the baseline IL-6 levels, the relative risk for development of biochemical progression was 1.9 (95% CI, 1.088–3.281; *P*=0.024). Similarly, for TNF-*α* the relative risk was slightly higher at 2.1 (95% CI, 1.144–4.12; *P*=0.018).

## DISCUSSION

Elevated serum levels of specific cytokines have been previously described in prostate cancer benign prostate hyperplasia specimens and in prostate cancer cell lines (Chen *et al*, 2000; Mizokami *et al*, 2000).

Twillie *et al* (1995) showed elevated serum IL-6 levels in 47% of 73 patients with advanced hormone refractory prostate cancer and suggested that this cytokine mediated morbidity in patients with metastatic disease. Likewise, Drachenberg *et al* (1999) reported high concentrations of IL-6 in men with hormone refractory prostate cancer compared to normal controls, benign prostatic hyperplasia, prostatitis and localised or recurrent disease.

We report elevated serum levels of the cytokines IL-6 and TNF-*α* in patients with prostate cancer as compared with controls. There was a strong association between the serum levels of the cytokines, disease stage and the presence of metastatic disease. TNF-*α* was significantly elevated in patients with metastatic cancer compared with the two groups of patients with localised disease and controls, and was correlated with increasing PSA. In contrast, IL-6 was correlated with tumour burden independently of PSA.

In patients with progressive disease, both IL-6 and TNF-*α* were elevated at the point of biochemical progression and further raised at symptomatic progression. These cytokines may therefore provide additional markers to PSA that reflect the activity of prostate cancer.

Our results confirm the findings of previous studies and add to the literature by documenting the significance of IL-6 and TNF-*α* as predictors of progressive disease.

We postulate that serum IL-6 and TNF-*α* levels might be useful prognostic markers with regard to progression to biochemical or symptomatic relapse based on the results of serial measurements. To our knowledge, this is the first time in the reported literature that monitoring of serial serum concentrations of these cytokines has been suggested as possible indicators of disease progression. However, the multivariate regression analysis failed to confirm these cytokines as significant independent prognostic variables for patient survival.

IL-6 is known to promote the proliferation and metastatic potential of cancer cells.

This cytokine has been characterised as a prostate exocrine gene product that interacts with its receptor in prostate cells, regulating proliferation and differentiation, and in prostate cancer cell lines activates androgen receptor (AR).

IL-6 signalling is mediated through the Janus kinase (JAK), signal transducer, activator of transcription 3 (STAT3) and mitogen-activated protein kinase (MAPK), which induces AR-mediated gene activation in prostate cancer (Hobisch *et al*, 1998; Lou *et al*, 2000; Spiotto and Chung, 2000). Several *in vitro* studies on prostate cancer cell lines suggest that prostate cancer cells themselves are secreting a major portion of IL-6, although other sources cannot be excluded. As prostate cancer cells also express high levels of IL-6 receptor, IL-6 may elicit both paracrine and autocrine responses. Thus, during the progression of prostate cancer to the hormone refractory phenotype, IL-6 undergoes a functional transition from paracrine growth inhibitor to autocrine growth stimulator (Hobisch *et al*, 1998; Deeble *et al*, 2001). These androgen-insensitive cells are also TNF-*α* insensitive. This is attributed to the constitutive activation of antiapoptotic genes, since TNF-*α* activates cell survival and cell death mechanisms simultaneously and can influence cell growth by apoptotic and nonapoptotic mechanisms (Muenchen *et al*, 2000).

However, many signalling pathways through which IL-6 and TNF-*α* mediate their effects have not been defined yet. A better understanding of how these cytokines contribute to the pathophysiology of prostate cancer would provide new targets for therapeutic intervention.

In conclusion, viewed in the context of similar observations made for other cancers, these data further support a relationship between elevated IL-6 and TNF-*α* levels and metastatic prostate cancer. Both cytokines correlate with the extent of disease and should be monitored in conjunction with other disease indicators. Although it is possible that these cytokines have a casual role in cancer progression, it is also of course clear that they may have a bystander role in malignancy.
