# Serum levels of IL-6 and TNF-*α* correlate with clinicopathological features and patient survival in patients with prostate cancer

**DOI:** 10.1038/sj.bjc.6602115

**Published:** 2004-09-10

**Authors:** V Michalaki, K Syrigos, P Charles, J Waxman

**Correction to:** *British Journal of Cancer* (2004) **90**, 2312–2316. doi:10.1038/sj.bjc.6601814

Due to an error, the second author's name was omitted from the above paper. The correct author listing is shown below:

V Michalaki, M Odontiadis, K Syrigos, P Charles and J Waxman

Also, in the same paper, part of the labelling in Figures 1

**Figure 1:**
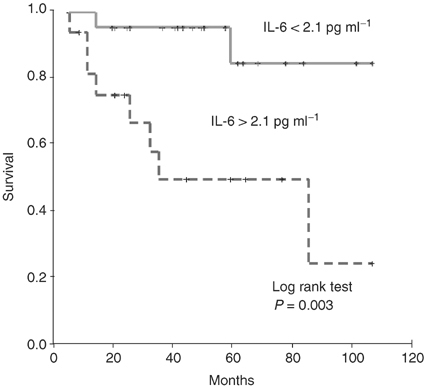
Disease-specific survival in patients with localised prostate cancer, stratified into groups above or below the cutoff IL-6 level of 2.1 pg ml^−1^.

and 2

**Figure 2:**
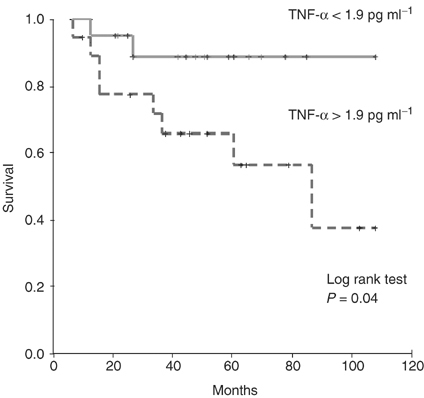
Disease-specific survival in patients with localised prostate cancer, stratified into groups above or below the cutoff TNF-*α* level of 1.9 pg ml^−1^.

is incorrect. The correct figures are reproduced below:

